# Extraction of Germanium from Low-Grade Germanium-Bearing Lignite by Reductive Volatilization

**DOI:** 10.3390/ma16155374

**Published:** 2023-07-31

**Authors:** Rengao Yang, Weifeng Song, Shuai Rao, Jinzhang Tao, Dongxing Wang, Hongyang Cao, Zhiqiang Liu

**Affiliations:** 1 School of Environmental Science and Engineering, Guangdong University of Technology, Guangzhou 510006, China; 2 Institute of Resources Utilization and Rare Earth Development, Guangdong Academy of Sciences, Guangzhou 510650, China; 3 State Key Laboratory of Separation and Comprehensive Utilization of Rare Metals, Guangzhou 510650, China

**Keywords:** germanium, volatilize, reduction, lignite

## Abstract

Germanium (Ge) as an important strategic metal is widely used in many modern-technology fields such as optical fiber and thermal solar cells. In this study, the volatilization behavior of Ge from low-grade germanium-bearing lignite was investigated in detail through reductive volatilization. The results indicated that temperature and air flow rate in the semi-closed roasting system played a significant role in the process. The optimal volitation efficiency of Ge reached 98% at 1100 °C for 2 h with air flow rate of 0.7 L/min in a maffle furnace, respectively. Under optimal conditions, the contents of Ge lowered to 30 ppm in the roasting residue. Analysis of the enriched ash yielded 71,600 ppm for Ge. Chemical phase analysis of the Ge in the enrichment ash showed that soluble Ge accounted for 82.18% of the total Ge, which could be viewed as an excellent material for Ge extraction by chlorinated distillation.

## 1. Introduction

Germanium is a grayish-white semiconductor element with electrical properties intermediate between those of metals and insulators, as well as physicochemical properties similar to silicon and tin. Because of its unique physicochemical properties and compounds, it is used in a variety of industries and modern-technology fields [1,2,3]. Meanwhile, considering the lack of alternative materials and the risk of supply, germanium has been listed as a strategic metal in many countries including China, America, Japan and Europe [4]. Elshkaki et al. [5] expected that the average annual demand for germanium is expected to become 2130% of 2010 production levels by 2050.

Germanium is scarce in nature, with an average abundance of only 1.5 ppm in the earth’s crust [6]. As a typical scattered element, it is generally distributed in lead and zinc mines (about 7000 tons) and coal mine (about 112,000 tons) deposits [7,8,9,10]. Therefore, germanium is mainly recovered as a by-product from lead and zinc mines and coal mines. Currently, approximately 50% of germanium is extracted from germanium coal in China.

At present, the main methods for extracting germanium from coal fly ash contained acidic leaching [11,12,13], biotechnologies [14] and volatile enrichment by pyrometallurgy [15]. Chen et al. [16] used acid to directly leach germanium from germanium coal. The results showed that, during direct acidic leaching, the leaching efficiency of germanium was as low as 83.62% (initial content of 17,300 ppm). It can be attributed to the fact that germanium in the coal appeared in the form of organic bonding [17]. Su et al. [18] used biotechnologies to recover germanium from critical raw materials, offering the advantages of environmental friendliness, low production costs and the ability to process complex minerals. However, biotechnologies are considered to be sensitive to inhibitors and show too-low kinetics. The above factors have limited the application of biotechnologies. Combustion volatilization is a promising and alternative approach for extracting germanium from coal fly ash. Zhang et al. [19] demonstrated the use of a vacuum reduction combustion process resulting in a 94.64% recovery ratio of germanium from coal fly ash with a germanium content of 6158 ppm.

High-grade germanium coal has been used for industrial production by some companies in Yunnan and Inner Mongolia, China, but low-grade germanium coal remains unexploited due to economic reasons [20,21]. However, with frequent mining over a long period of time, the reserves of high-quality coal have rapidly diminished [22,23]. Lignite is a typical low-quality coal. Although the grade of germanium (ranging from 30 ppm to 200 ppm) in lignite is poor, the large reserves of lignite will make it a major raw material for germanium extraction in the future [24,25]. There is an urgent need to develop better technology to utilize the lignite. In this study, the low-grade germanium-bearing lignite from Wulantuga Mine, Inner Mongolia was selected as the research object. During the combustion process, the main influential factors on the volatilization behaviors of germanium were investigated in detail. At the same time, various combustion residues and concentrate were characterized using ICP-OES, XRD and SEM-EDS.

The objective of this study was to achieve the separation of carbon and germanium while enriching soluble germanium to provide the basis for subsequent purification by chlorinated distillation.

## 2. Materials and Methods

### 2.1. Materials

This study was conducted using low-grade germanium-bearing lignite from Inner Mongolia (longitude 115°30′–116°35′ E, latitude 43°45′–44°18′ N). Particle size distribution of the lignite by Laser Scattering Particle Size Distribution Analyzer. ICP-OES, XRD and SEM-EDS were used to characterize the content, phase and morphology of the main elements in the lignite, and it was analyzed industrially according to the national standard GB/T 30732-2014.

The results of the industrial and chemical analysis are shown in Table 1. By ICP-OES analysis, the content of germanium in the lignite was 190 ppm. In addition, a relatively high content of S (above 2.87 wt.%) and Si (above 3.7 wt.%) was detected in the lignite.

**Figure 1:**
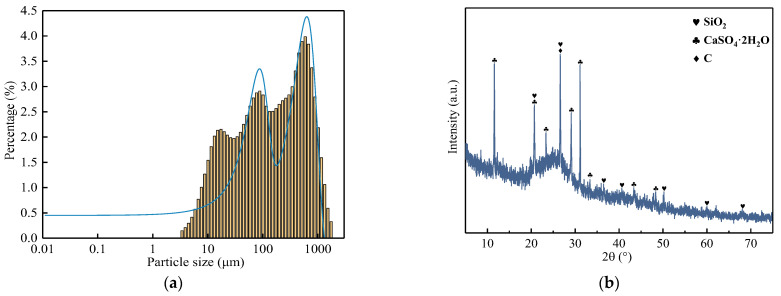
Particle size distribution (**a**) and XRD pattern (**b**) of the lignite.

**Figure 2:**
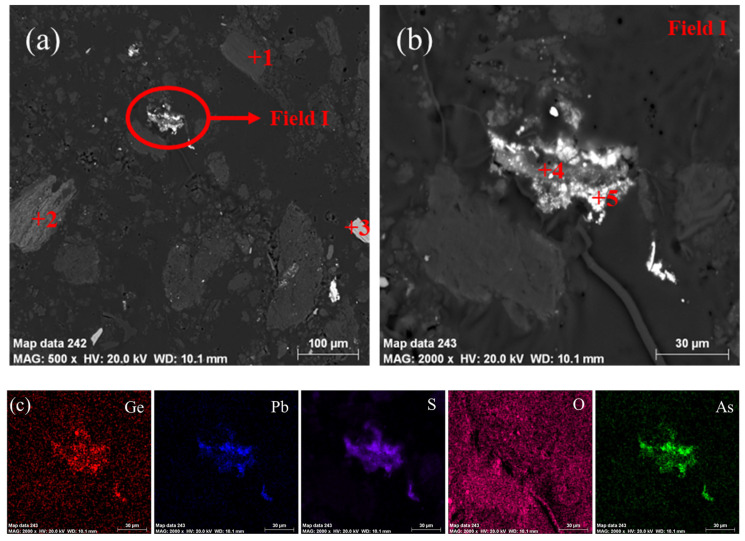
(**a**) SEM images of the lignite (**b**) SEM images of field I and (**c**) element mapping of field I.

The particle size distribution and chemical phase analysis of the lignite was carried out as detailed in Figure 1. Since the experiment was carried out using untreated lignite, its particle size distribution was not uniform, and according to the measurements, its D50 was 130.5894 μm, as shown in Figure 1a. From Figure 1b, the main phase of the lignite was C, SiO_2_ and CaSO_4_·2H_2_O. Figure 2 shows SEM images of the germanium-containing lignite. Based on EDS results from Table 2, spot 4 and spot 5 contained an especially high content of germanium. Moreover, germanium presented a close relationship with As, Pb and S, as shown in Figure 2c. Spot 1 represented a C-rich particle containing low contents of S. Spot 2 was viewed as silicon dioxide owing to high contents of Si and O. Moreover, Spot 3 was recognized as calcium sulfate because of high contents of Ca, S and O. This is consistent with the XRD analysis.

### 2.2. Experiment

In order to research the volatile processes of germanium, combustion experiments were designed in a muffle furnace (KSL-1200X-M) under different conditions. The raw materials were placed in an alumina crucible and transferred to the middle of the muffle furnace. After the roasting process, the roasting residue was weighed. An acidic mixture (H_3_PO_4_: HF: HNO_3_ = 1:1:2) was used to dissolve the residue, and the solution was used to analysis the content of germanium by ICP-OES. The volatilization efficiency of germanium was calculated through the determination of the content of germanium before and after roasting. The volatilization efficiency of germanium is calculated from Equation (1) [26] as follows:

(1) $$\eta_{Me}=\frac{m_{1}\times wt_{1}-m_{2}\times wt_{2}}{m_{1}\times wt_{1}}\times 100\%$$

where *η_Me_* represent the volatilization rates of germanium; *m*_1_ and *m*_2_ refer to the mass of the material and the corresponding combustion residue, respectively; and *wt*_1_ and *wt*_2_ are the contents of the material and the corresponding combustion residue, respectively.

**Figure 3:**
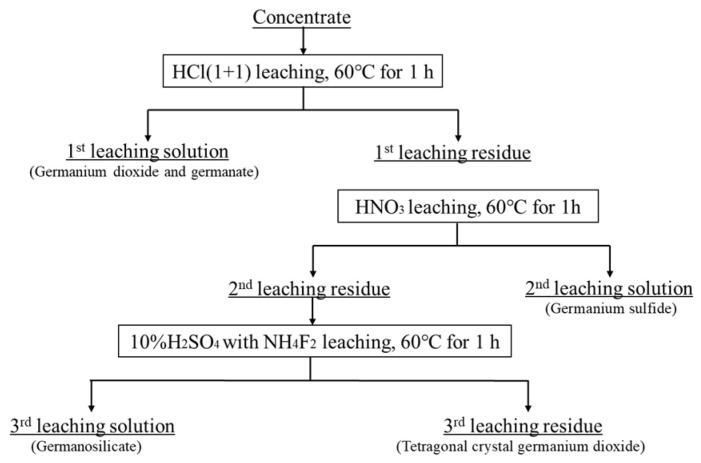
Technological route for the chemical physical phase analysis of germanium.

In addition, the germanium in the concentrate was studied according to the properties of the different phases of germanium. The technological route of the analysis is illustrated in Figure 3.

### 2.3. Characterization and Analysis

The residue and concentrate obtained from aforementioned experiments were digested in an acidic mixture in a heating panel (250 °C for digesting 1 h). The digestion solution was filtered through a paper membrane and finally made up to 100 mL using ultrapure water. The concentration of germanium was determined using inductively coupled plasma optical emission spectrometry (ICP-OES, Spectro ARCOS MV, Kleve, Germany) and based on the spectral line Ge209.426. X-ray diffraction (XRD, Rigaku TTR-III, Tokyo, Japan) and scanning electronic microscopy using energy dispersive spectrometer (SEM-EDS FEI Quanta 650, Bruker Quantax, Billerica, MA, USA) were used to characterize the morphology of the residue and concentrate at optimum roasting conditions.

## 3. Results and Discussion

### 3.1. Thermodynamic Analyses

#### 3.1.1. Chemical Reaction Equations for Ge–C–O

**Figure 4:**
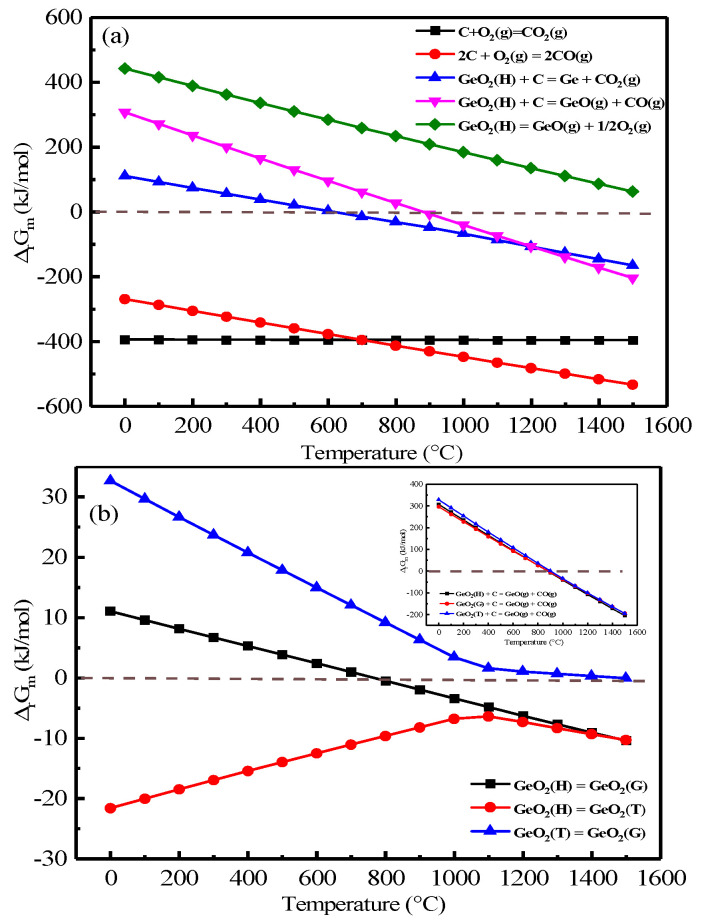
Main chemical equations and Gibbs free energies for Ge–C–O (**a**,**b**).

During the combustion process, the possible reaction for germanium is listed in Table 3. Meanwhile, the Gibbs free energy was calculated based on HSC 6.0 and the result is shown in Figure 4.

It was evident from Figure 4a that hexagonal germanium dioxide (GeO_2_(H)) had difficulty in decomposing into GeO(g) via Number 3–5 in the 1500 °C range without the involvement of carbon in the reaction. In the presence of carbon, GeO_2_(H) was reduced to Ge and GeO(g) at 600 °C and 900 °C, respectively, and, as the reaction temperature increased, decomposition to GeO(g) was more prone to occur when the temperature reached 1100 °C. From Figure 4b, it can be seen that diverse crystalline forms of GeO_2_ can be transformed into each other in different temperature ranges. Among them, GeO_2_(H) can be spontaneously converted to tetragonal germanium dioxide (GeO_2_(T)). GeO_2_(H) and GeO_2_(T) can only be converted to glassy germanium dioxide (GeO_2_(G)) under high-temperature conditions. Additionally, the carbon thermal reduction of GeO_2_(T) was the most challenging among the carbon thermal reactions of the varied crystalline GeO_2_ types.

#### 3.1.2. Equilibrium Compositions Diagram of Ge–C–O Systems

**Figure 5:**
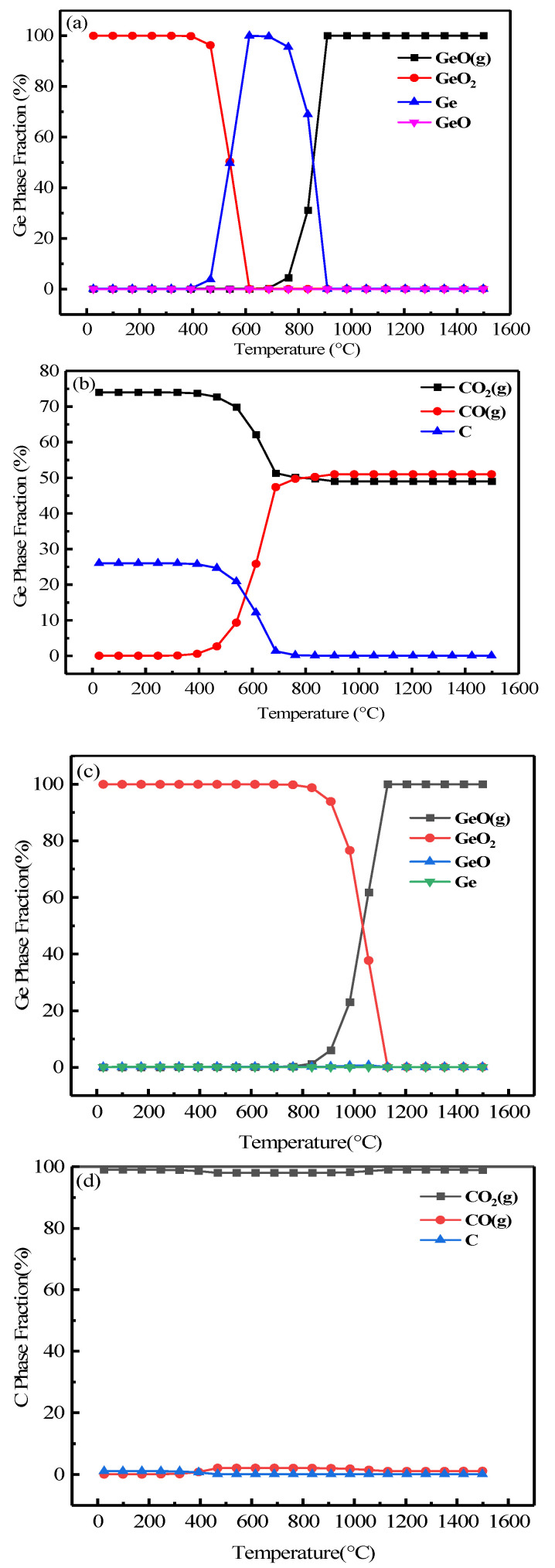
Equilibrium compositions diagram of the Ge–C–O system [(**a**,**b**) (n(O_2_)/n(C) = 0.75); (**c**,**d**) (n(O_2_)/n(C) = 1)].

As shown in Figure 5, the equilibrium compositions of germanium in the lignite were plotted for the Ge–C–O systems using the “Equilibrium Composition” module in HSC 6.0 in order to clarify the equilibrium compositions of germanium at different temperatures and systems.

By fixing the amount of carbon, the amount of oxygen was changed, which in turn created a distinct atmosphere. It has been calculated that germanium dioxide can be reduced to germanium metal at a low temperature (<900 °C) when the combustion environment is a potent reducing atmosphere [n(O_2_)/n(C) = 1]. When the combustion environment was a potent oxidizing atmosphere [n(O_2_)/n(C) = 2] and the temperature was below 1200 °C, germanium was mainly present as a crystal type of GeO_2_. Temperatures in excess of 1500 °C were required if GeO(g) crystals were to be formed. Clearly, germanium was difficult to reduce to GeO(g) for volatilization, either in a potent reducing nor oxidizing atmosphere. However, as can be seen in Figure 5a–d, in case the combustion environment was a suitable reducing atmosphere [(n(O_2_)/n(c) = 0.75), (n(O_2_)/n(c) = 1)], it was possible to achieve complete volatilization of GeO(g) crystal by adjusting the CO concentration and temperature fluctuations within a certain range. It was clear that complete volatilization of GeO(g) can be achieved at temperature of 900 °C when the CO ratio was approximately 50% (Figure 5a,b). The same result can be realized by increasing the temperature to 1100° C in case the CO ratio was approximately 1% (Figure 5c,d).

#### 3.1.3. Primary Combustion Thermogravimetric Analysis

**Figure 6:**
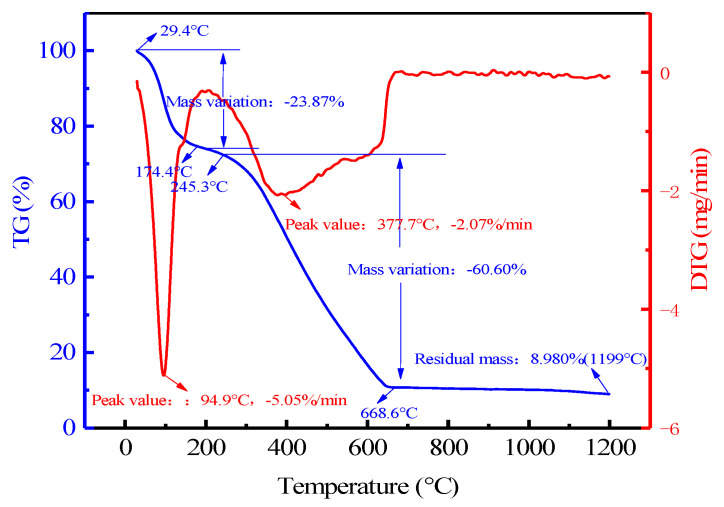
TG–DTG curve of reduction reaction of lignite.

Figure 6 shows the TG–DTG curve of the lignite combustion process. The measurements were performed under mixed air atmosphere. Samples of 8.0223 mg were placed in an alumina crucible and heated from room temperature to 1200 °C with a heating rate of 10 °C/min.

Table 4 illustrates the TG–DTG data. According to the data, we can find that before 174.4 °C the weight loss of sample was about 23.87%, accounting for 26.23% of the total weight loss. The weight loss at this stage was close to the content of inherent moisture in the lignite (26.32%), which could be attributed to the water evaporates from the lignite. The second stage of weight loss occurred in the range of 174.4–668.6 °C. In this stage, the weight loss rate was 60.60%, accounting for 66.58% of the total weight loss. It can be concluded from Figure 6 that the weight was a sharp decline starting from about 245.3 °C. The explanation was that the combustion of carbon in the lignite and the weight loss in this stage were slightly higher than the content of fixed carbon (43.37%). With the heating temperature exceeding 668.6 °C, the weight loss of lignite was more moderate. The mass loss at this stage was only 6.55%, accounting for only 7.20% of the total weight loss, which might be due to the decomposition of small amounts of carbonate and sulfate, as well as oxide in the lignite; therefore, weightlessness was caused by the reaction of the sulfate, oxide and coke. The reduction reaction of the GeO_2_ should occur in this stage, for which the following chemical reactions are involved:

(2) $$2{\mathrm{CaSO}}_{4\;}+C=2\mathrm{CaO}+2{\mathrm{SO}}_{2}+{\;\mathrm{CO}}_{2}$$

(3) $${\mathrm{GeO}}_{2}+C=\mathrm{GeO}+\mathrm{CO}$$

### 3.2. Combustion Experiments

Equilibrium composition diagrams demonstrated that temperature and roasting system exerted a significant effect on the volatilization behavior of germanium. In this research, the effects of roasting system, air flow rate, temperature, heating time and heating rate on the volatilization efficiency of germanium were investigated in detail.

#### 3.2.1. Effect of Roasting System

**Figure 7:**
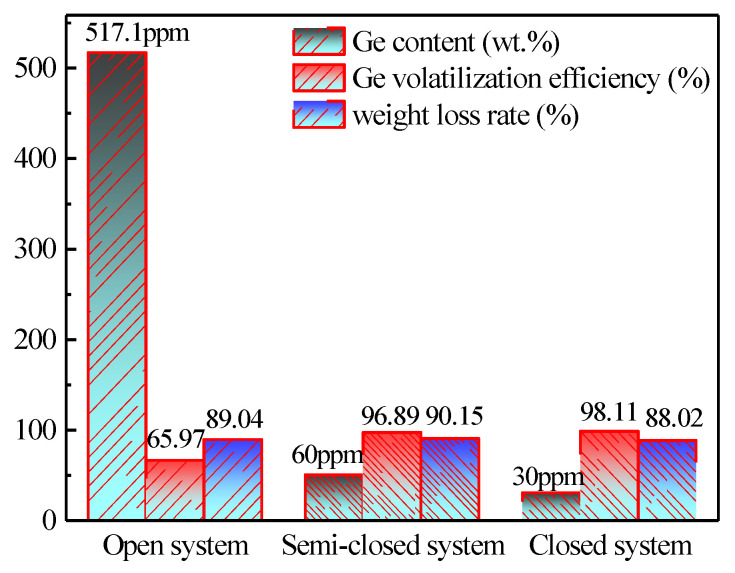
Effect of roasting system on the volatilization of germanium in lignite (Operation conditions: temperature of 1100 °C, air flow rate of 0.7 L/min, heating rate of 8 °C/min, heat time of 180 min).

The relationship between the volatilization rate of germanium and roasting system is shown in Figure 7. It shows that the roasting system of lignite had a significant influence on the volatilization efficiency of germanium. The germanium content in the residue reached 517.1 ppm when the sample was burned in the muffle furnace and the furnace door was fully opened, and the volatilization efficiency was only 65.97%; when the semi-closed system was adopted (muffle furnace burning, furnace door closed, air was introduced), the germanium content was reduced to 60 ppm from 517.1 ppm in the residue, and the volatilization efficiency reached 96.89%. When a completely closed system (tube furnace combustion, air was introduced) was studied, the germanium content in the residue was reduced to 30 ppm and the volatilization efficiency jumped to 98.11% from 65.97%. This can be explained by the fact that when the system was fully open, because of the rapid exposure to enough oxygen, a part of the generated GeO turned into non-volatile GeO_2_ and sank in the slag, resulting in a low germanium volatilization rate, whereas it was difficult for this to happen in semi-closed or closed systems. In order to make the experimental process simulate industrial production as closely as possible, the subsequent exploration will be carried out in a semi-closed system (muffle furnace burning, furnace door was closed, air was introduced).

#### 3.2.2. Effect of Air Flow Rate

**Figure 8:**
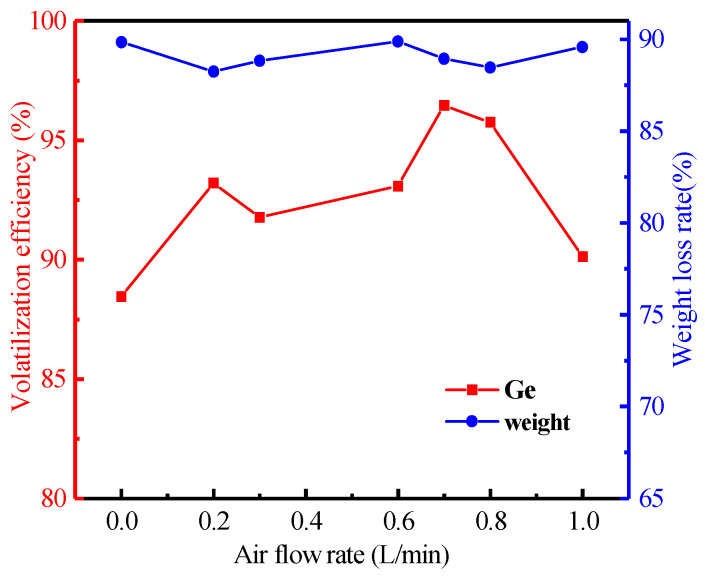
Effect of air flow rate on the volatilization of germanium in lignite (Operation conditions: temperature of 1100 °C, heating rate of 8 °C/min, heat time of 120 min).

It is possible to control the combustion atmosphere by varying air flow rate. Figure 8 shows the relationship between volatilization efficiency of germanium and air flow rate. The effect of air flow rate on the volatilization efficiency was investigated in the range from 0 to 1.0 L/min. Figure 8 showed that with the increase in air flow rate from 0 to 0.7 L/min, the volatilization efficiency of germanium increased from 88.46% to 96.46%. However, the volatilization efficiency of germanium was reduced when the air flow rate continued to increase to 1.0 L/min. It can be explained that the increased air flow rate was beneficial to the combustion of carbon in the lignite and the formation of easily volatile GeO(g) when the air flow rate was less than 0.7 L/min. However, when the air flow rate was in the range from 0.7 to 1.0 L/min, the volatilization efficiency of germanium was reduced because the crystal form of germanium changed as the air composition changed. The volatilization effect of germanium with different crystal forms was different, so the volatilization rate of germanium also changed with the air flow rate. This indicated that germanium may have formed compounds such as GeO_2_ and CaGeO_3_, which were difficult to volatilize.

#### 3.2.3. Effect of Temperature

**Figure 9:**
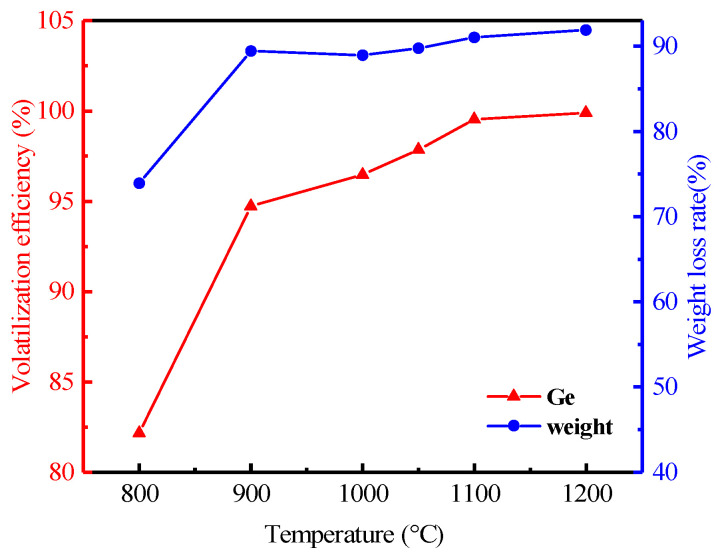
Effect of temperature on the volatilization of germanium in lignite (Operation conditions: heating time of 120 min, the air flow rate of 0.7 L/min, heating rate of 8 °C/min.).

With the temperature increasing from 800 to 1200 °C, the effect of temperature is shown in Figure 9. It shows that the volatilization efficiency of germanium was significantly increased with the increase in temperature. The evaporation of metallic germanium and its oxides was based on their saturation vapor pressure and a sharp increase appears at 1100 °C, where the volatilization rate of germanium jumped to 99.53% from 82.14%. Therefore, the optimal heating temperature for the volatilization efficiency of germanium was chosen to be 1100 °C.

#### 3.2.4. Effect of Heating Time

**Figure 10:**
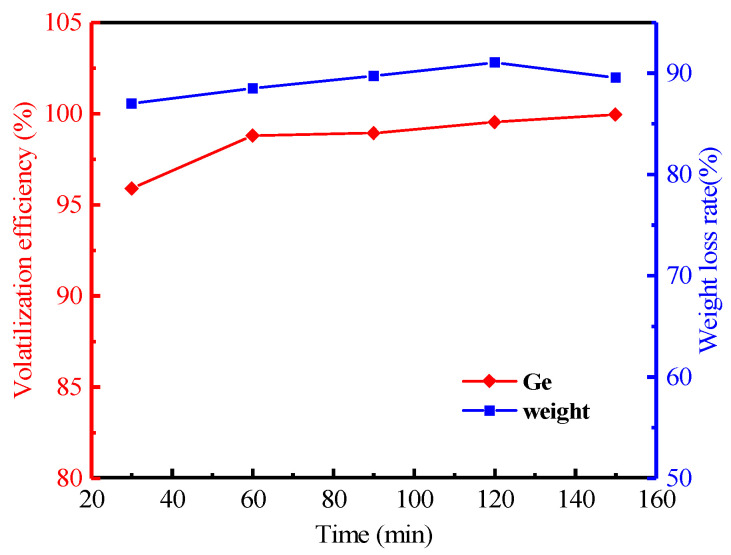
Effect of heating time on the volatilization of germanium in lignite (Operation conditions: temperature of 1100 °C, the air flow rate of 0.7 L/min, heating rate of 8 °C/min.).

The relationship between volatilization efficiency of germanium and time is shown in Figure 10, which shows that the germanium’s volatilization efficiency can reach up to 99.53% at 120 min. The continued extension of the heating time had no significant effect for germanium. This shows that the heating time had a small effect on the volatilization of germanium at the right temperature. Therefore, the optimal reaction time was 120 min for the volatilization efficiency of germanium substances from the lignite.

#### 3.2.5. Effect of Heating Rate

**Figure 11:**
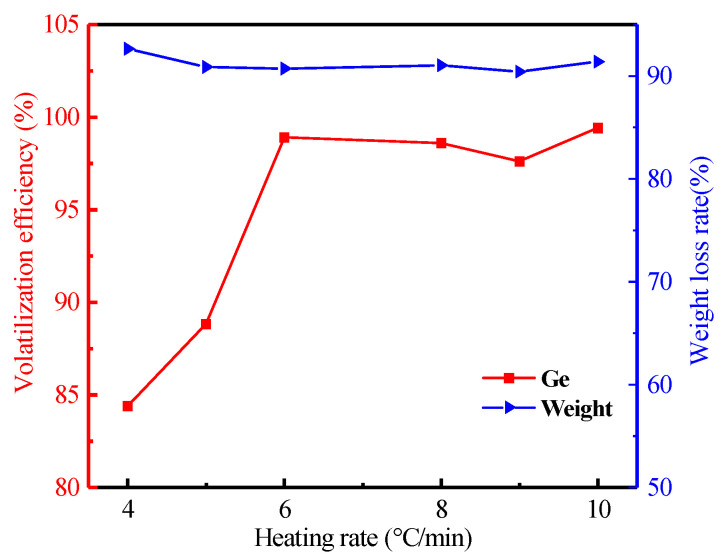
Effect of heating rate on the volatilization of germanium in lignite (Operation conditions: temperature of 1100 °C, the air flow rate of 0.7 L/min, heating time of 120 min).

With the heating rate increasing from 4 to 10 °C/min, the effect of heating rate is shown in Figure 11. This figure shows that the germanium’s volatilization efficiency can reach up to 98.60% at 8 °C/min. It does not present the trend that the volatilization efficiency of germanium increased with the increasing heating rate. The result indicated that the effect of heating rate for volatilization efficiency was not significant. Therefore, the optimal heating rate was considered to be 8 °C/min for the volatilization efficiency of germanium.

### 3.3. Analysis of Residues and Concentrate

The optimal parameters of volatilization efficiency of germanium from the lignite were obtained through a series of experiments. The optimal parameters were at a temperature of 1100 °C, heating time of 120 min, heating rate of 8 °C/min and air flow rate of 0.7 L/min. In order to collect concentrate, the experiments are performed in a closed system (in a tube furnace) according to the optimal parameters.

#### 3.3.1. Characterization of the Combustion Residue

**Figure 12:**
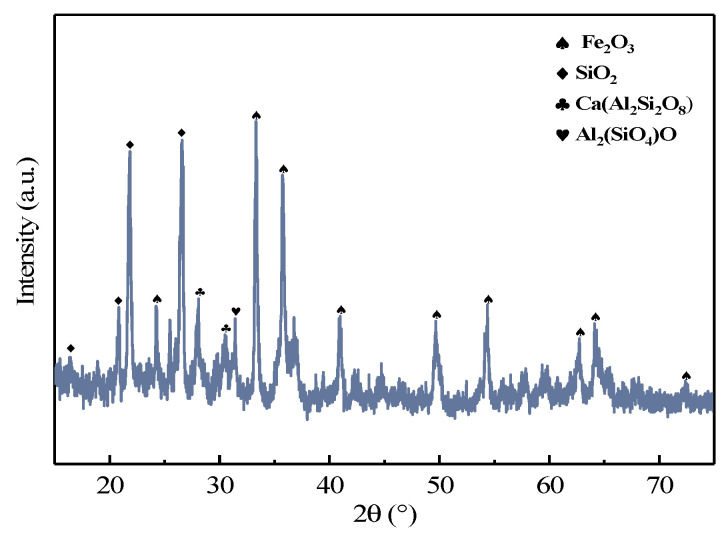
XRD patterns of the combustion residue.

**Figure 13:**
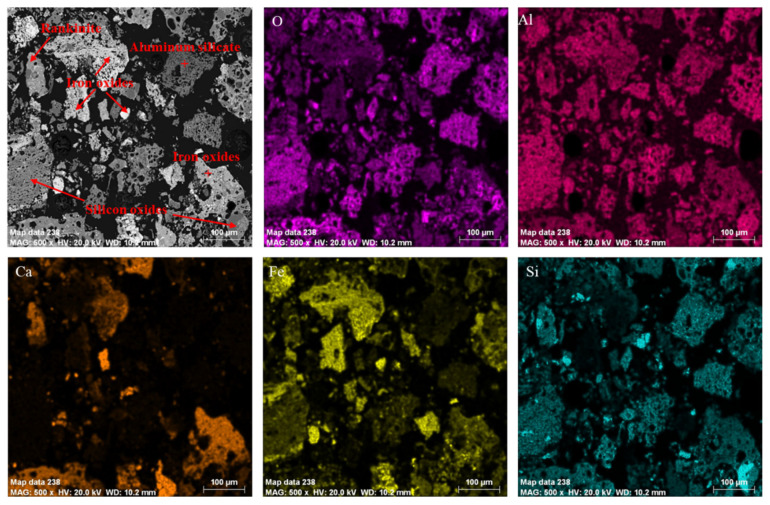
SEM analysis of the combustion residue and its main elemental surface distribution.

The chemical composition of the combustion residue is shown in Table 5. According to Table 5, germanium in the residue was reduced to 30 ppm, which meant that germanium in the lignite was well volatilized. Meanwhile, we analyzed the morphology of the residue with XRD and SEM. As can be seen from Figure 12, the combustion residue was mainly composed of iron oxide, silica dioxide, calcium aluminum silicate and other phases. According to Figure 13, the combustion residue had a relatively simple physical phase, mainly iron oxides, silicon oxides, rankinite and aluminum silicate. However, neither XRD nor SEM could find a physical phase of the germanium, again indicating that the germanium was volatilized quite thoroughly.

#### 3.3.2. Characterization of the Combustion Concentrate

**Figure 14:**
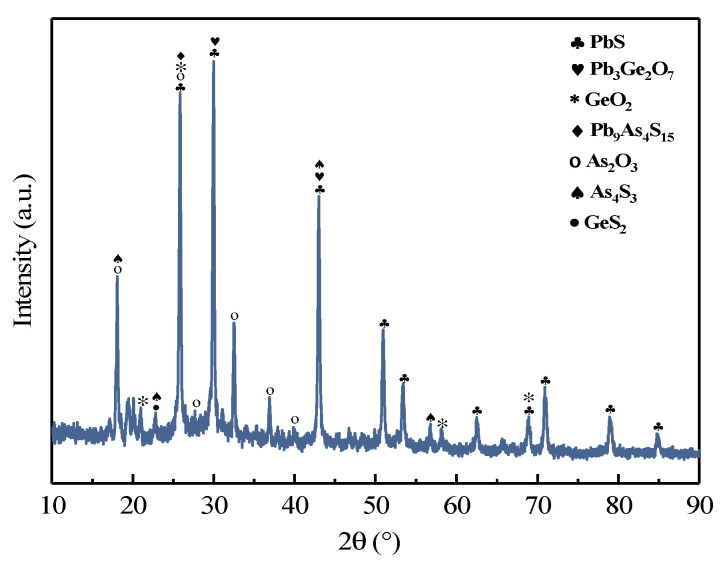
XRD patterns of the combustion concentrate.

**Figure 15:**
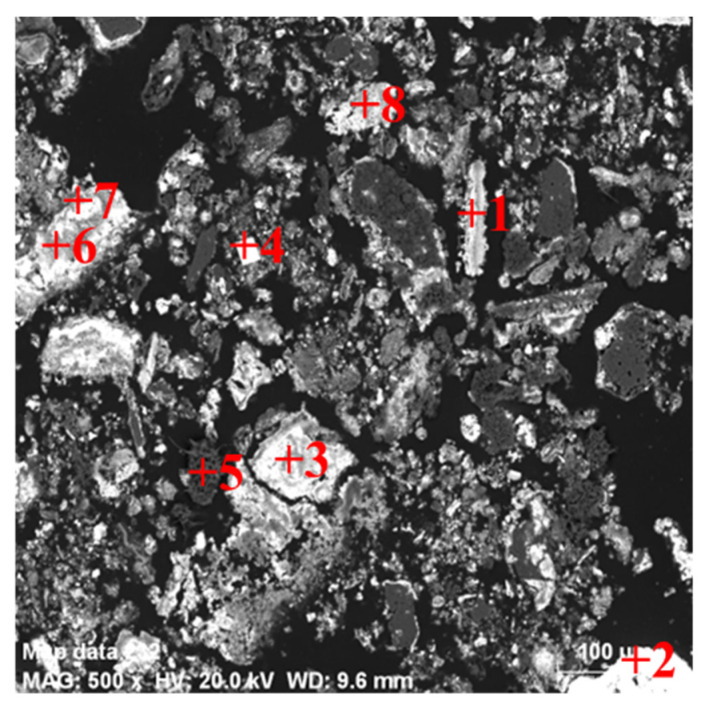
SEM-EDS analysis of the combustion concentrate.

Table 6 shows the chemical composition of the combustion concentrate. According to Table 6, germanium was enriched to 7.16% and concentrate products also contained a certain amount of As, as well as significant amounts of Pb and S. In addition, chemical phase analysis of the germanium in the concentrate was carried out and the results were shown in Table 7. The analytical results showed that the content of soluble (germanium oxide, germanate, germanium sulfide) and insoluble (germanosilicate, tetragonal germanium dioxide) germanium was 82.18% and 17.82%, respectively. Meanwhile, the morphology of the concentrate was analyzed by XRD and SEM-EDS. As can be seen from Figure 14, the main phase of germanium was lead germinate, and besides lead germinate in the concentrate, the concentrate also contained arsenic trioxide, lead sulfide, silica, etc. SEM-EDS analyses for the concentrate are shown in Figure 15. It can be seen that the obtained concentrate presented an irregular morphology. EDS results from Table 8 demonstrated that the concentrate was mainly composed of Pb, Ge, As, O and S. According to the results of EDS analysis, the germanium content in spot 1, 6 and 8 was approaching 60%, 20% and 15%, respectively. In addition, there were higher levels of As, O, and S. This indicated that germanium and arsenic did not only volatilize in a unique form of GeO and As_2_O_3_, respectively. Germanium and arsenic were also volatilized as sulfides, which was consistent with the results of the chemical phase analysis. The lead content in spot 2 exceeded 98%, which indicated that part of the lead was evaporated in the form of lead elemental. In spots 3, 4 and 7, the contents of Pb and S were high and the O content was low; this indicated that lead could be volatilized with PbS. The chemical phase analysis revealed only 0.62 wt.% germanosilicate in the combustion concentrate, which meant that the silicon might have been volatilized during the combustion process in different forms, such as silicon fluoride (spot 5).

## 4. Conclusions

In this study, the recovery process of germanium was investigated from low-grade germanium-bearing lignite in semi-closed or closed systems. The feasibility of recycling germanium from the lignite was verified by experiments and thermodynamic analysis. The optimal parameters of volatilization rates of germanium from the lignite were obtained through a series of laboratory scale experiments. The optimal parameters were at a temperature of 1100 °C, heating time of 120 min, heating rate of 8 °C/min and air flow rate of 0.7 L/min. Under the optimal parameters, the volatilization rate of germanium exceeds 98%, and the germanium content is only 30 ppm in the residue. The concentrate was analyzed and it was found that the germanium content in the concentrates was as high as 7.16%; among them, the content of soluble (germanium oxide, germanate, germanium sulfide) and insoluble (germanosilicate, tetragonal germanium dioxide) germanium was 82.18% and 17.82%, respectively. Therefore, this technological process can realize the enrichment of germanium from germanium-bearing lignite, which lays the foundation for further purification using chlorinated distillation or leaching to subsequently obtain pure germanium.

## Figures and Tables

**Table 1:** Industrial and elemental analysis of the lignite (wt.%).

Sample		Lignite
Industrial analysis	M_ad_	26.32
	V_ad_	43.11
	A_ad_	13.52
	FC_ad_	43.37
Chemical analysis	Ge	0.019
	As	0.053
	C	34
	S	2.87
	Si	3.4
	Pb	<0.001

M_ad_: intrinsic moisture; V_ad_: volatile matter; A_ad_: ash; FC_ad_: fixed carbon.

**Table 2:** Chemical composition of the selected particle from Figure 2 (wt.%).

Spot	C	O	Si	Ge	Ca	Pb	S
1	44.64	35.54	0.21	0.3	-	-	4.02
2	-	30.41	20.42	-	-	-	2.83
3	-	45.57	-	-	32.51	-	21.69
4	-	44.91	0.27	4.04	0.03	15.35	24.52
5	-	20.28	-	2.53	0.07	62.95	8.63

**Table 3:** Possible reaction in the reduction process.

Reaction Equation	Number
C + O_2_(g) = CO_2_(g)	3-1
2C + O_2_(g) = 2CO(g)	3-2
GeO_2_ (H) + C = Ge + CO_2_(g)	3-3
GeO_2_ (H) + C = GeO(g) + CO(g)	3-4
GeO_2_ (H) = GeO(g) + 1/2O_2_(g)	3-5

**Table 4:** Mass losses of lignite at different temperatures range at a heating rate of 10 °C/min.

Reaction Stage	Temperature Rage (°C)	Weight Loss (%)	Total Weight Loss (%)	Percent of Weight Loss (%)
I	<174.4	23.87	91.02	26.23
II	174.4–668.6	60.60		66.58
III	668.6–1200	6.55		7.20

**Table 5:** Chemical composition of the combustion residue (wt.%).

Element	Ge	As	C	S	Si	Pb
Residue	0.003	0.082	0.042	0.98	11.57	<0.001

**Table 6:** Chemical composition of the combustion concentrate (wt.%).

Element	Ge	As	C	S	Si	Pb
Concentrate	7.16	6.88	-	12.51	3.28	25.79

**Table 7:** Result of chemical phase analysis of germanium (wt.%).

Chemical Phase	Content	Percent
germanium oxide, germanate	3.2127	44.87
germanium sulfide	2.6712	37.31
germanosilicate	0.0445	0.62
tetragonal germanium dioxide	1.2316	17.20
total	7.16	-

**Table 8:** Chemical composition of the selected particle from Figure 15 (wt.%).

Spot	O	F	Si	S	Ge	As	Pb
1	8.26	1.84	0.45	14.06	58.93	7.67	-
2	0.24	1.44	-	-	-	-	98.32
3	1.51	0.46	-	20	3.78	21.74	45.42
4	-	-	-	10.63	0.72	-	85.47
5	2.89	14.59	35.54	17.62	11.75	5.18	-
6	10.95	0.78	-	25.52	18.38	19.05	15.77
7	2.34	-	-	26.05	4.43	23.56	32.45
8	7.67	3.05	0.94	17.94	13.02	11.81	35.54

## Data Availability

The data of the study are available from the corresponding author upon reasonable request.
